# Supplementary material for: Estimating the generation time for influenza transmission using household data in the United States
Source: medRxiv. 2024 Aug 19:2024.08.17.24312064. Preprint. [Version 1] doi: 10.1101/2024.08.17.24312064 (PMC11370535; doi:10.1101/2024.08.17.24312064)
Supplement: Supplement 1 [file NIHPP2024.08.17.24312064v1-supplement-1.pdf]

medRxiv preprint doi: https://doi.org/10.1101/2024.08.17.24312064; this version posted August 19, 2024. The copyright holder for this preprint (which was not certified by peer review) is the author/funder, who has granted medRxiv a license to display the preprint in perpetuity. This article is a US Government work. It is not subject to copyright under 17 USC 105 and is also made available for use under a CC0 license.

Supplementary material

The incubation period distribution

The incubation period distribution was modeled using estimates for influenza A from a systematic review by Lessler et al. (Lessler, et al. 2009), with a mean of 1.55 days and a standard deviation (SD) of 0.66 days. These estimates were fitted to a gamma distribution to characterize the distribution of the incubation period (Supplemental Figure S1).

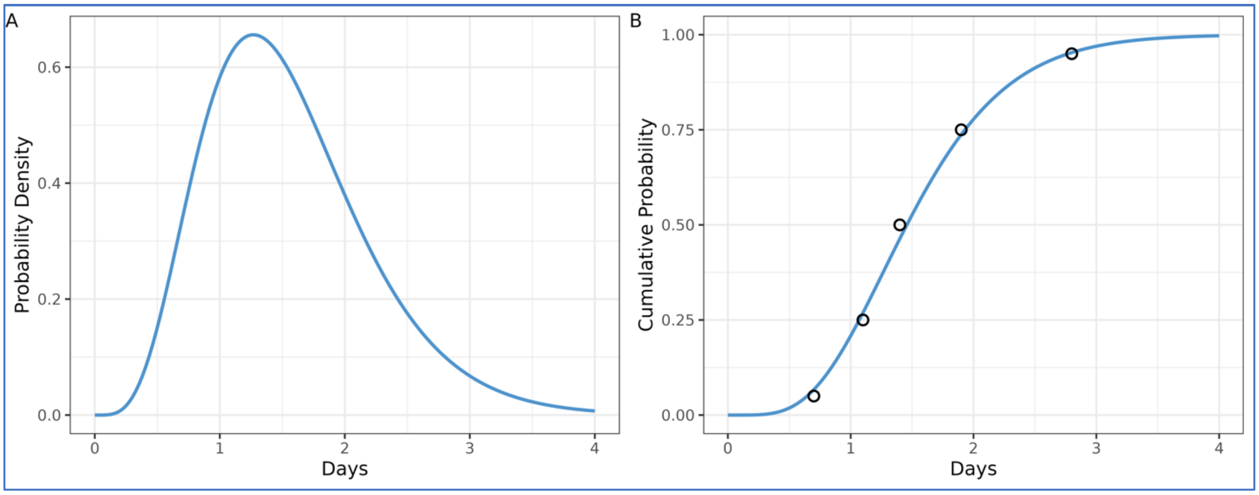

Figure S1. Incubation period distribution. The black circles and blue lines represent the data (Lessler, et al. 2009), and the (A) cumulative distribution function and (B) probability density function of a gamma distribution fitted to the data.

The observed household serial interval of single infection pairs

We found that the observed household serial interval, calculated without modeling, solely using data from households with single infection pairs (i.e., single primary case to single secondary case) and without potential transmission chains, had a mean of 3.7 days (and a SD of 2.3 days). This was longer than the mean intrinsic serial interval of 3.2 (95% CrI: 2.8-3.5) days when considering households of all sizes with all potential transmission chains (Table 2). This does not necessarily indicate that the intrinsic value was shorter than the realized household one. Rather, it is mainly due to the restriction of single infection pairs or mostly smaller household sizes of 2 members.

In the main text, we found slightly longer mean intrinsic and realized household generation times in smaller households compared to larger ones (Figure 1C and Supplemental Table S1). Larger households with more exposure and potential transmission chains could have a shorter interval, while smaller households could have a longer interval.

Specification of parameters for the mechanistic model

In the mechanistic model (Hart, Abbott, et al. 2022), two parameters, namely the ratio of the mean latent and incubation period and the mean symptomatic infectious period, were estimated directly (Supplemental Figure S2), while the proportion of transmission before symptomatic onset was calculated by weighting the pre-symptomatic period by the ratio of pre-symptomatic and symptomatic transmission rates and dividing it by the sum of the (pre-symptomatic and symptomatic) infectious periods. The mean latent and mean pre-symptomatic periods were calculated by dividing the incubation period by the ratio of mean latent and incubation period.

medRxiv preprint doi: https://doi.org/10.1101/2024.08.17.24312064; this version posted August 19, 2024. The copyright holder for this preprint (which was not certified by peer review) is the author/funder, who has granted medRxiv a license to display the preprint in perpetuity. This article is a US Government work. It is not subject to copyright under 17 USC 105 and is also made available for use under a CC0 license.

583 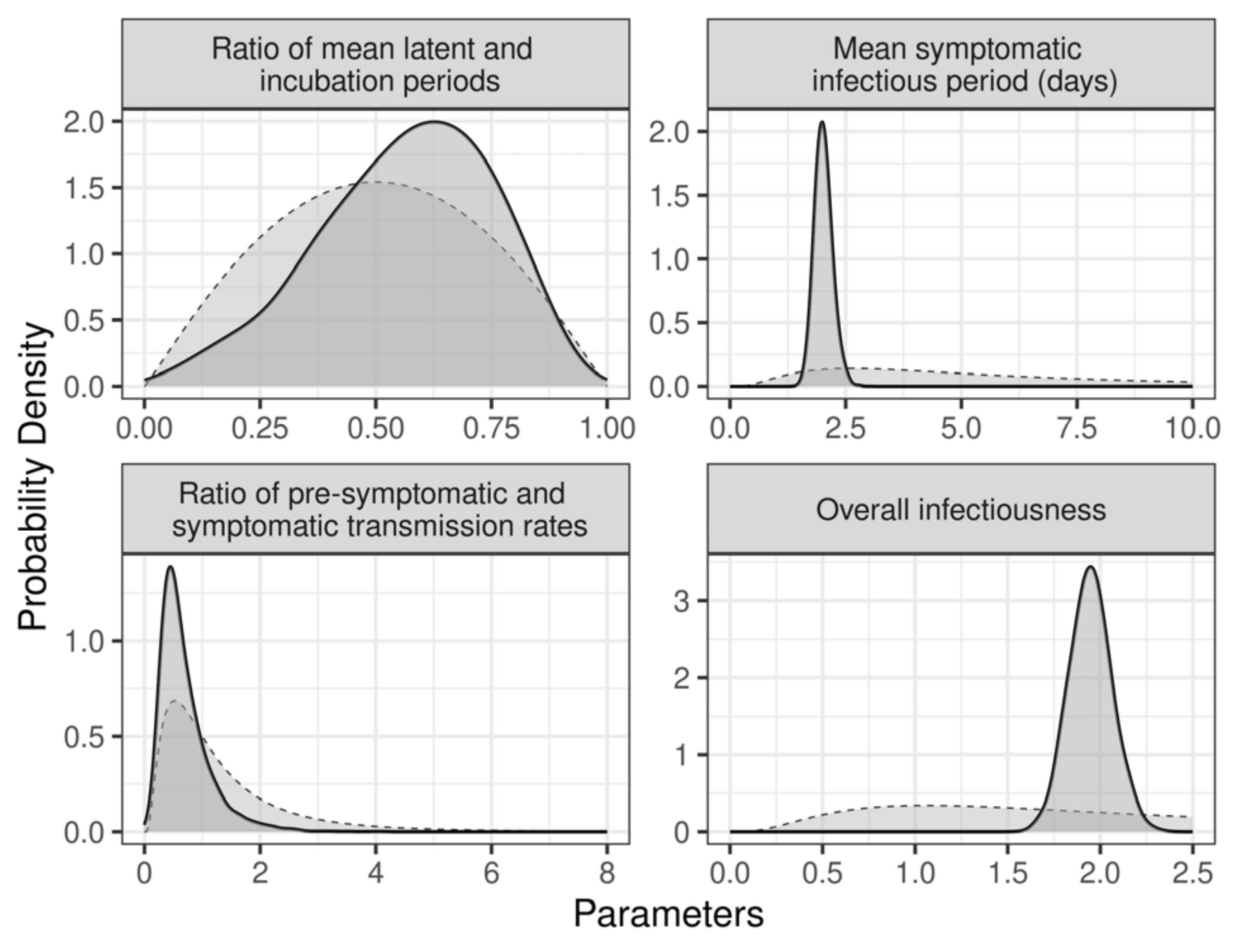

584 *Figure S2. Posterior and prior distributions of estimated parameters. Solid and dashed lines represent posterior and prior*
585 *distributions, respectively.*

586 ## Variability in estimates across data stratifications

587 Although the generation time or serial interval of influenza B may be longer than that of influenza A
588 (Levy, et al. 2013), this was not the case in our findings from the two seasons (Supplemental Table S1,
589 Figure S3 and S4). However, we note that the mean intrinsic generation time exhibited a wider credible
590 interval when using data exclusively from influenza B compared to influenza A, which likely reflects the
591 dominance of influenza A during the study timeframe and the smaller sample size of influenza B.

**Data stratifications**	**Mean intrinsic generation time (95% CrIs)**	**Overlapping index (%, compared to the primary analysis)**
All data excluding households with multiple co-primary cases (primary analysis in Table 1)	3.2 (2.9-3.6)	100
Season 2021/2022	3.3 (2.8-4.0)	71
Season 2022/2023	3.2 (2.8-3.6)	87
Influenza A	3.2 (2.9-3.6)	94
Influenza B	3.2 (2.3-4.5)	47

medRxiv preprint doi: https://doi.org/10.1101/2024.08.17.24312064; this version posted August 19, 2024. The copyright holder for this preprint (which was not certified by peer review) is the author/funder, who has granted medRxiv a license to display the preprint in perpetuity.
This article is a US Government work. It is not subject to copyright under 17 USC 105 and is also made available for use under a CC0 license.

Household size of 2 or 3	3.4 (2.9-4.0)	61
Household size of 4 or greater	3.1 (2.7-3.6)	74
All data including households with multiple co-primary cases	3.1 (2.7-3.4)	64

Table S1. The posterior mean (95% CrIs) of mean intrinsic generation time across seasons, virus types, household sizes, and with multiple co-primary cases. The incubation period, derived from influenza A, had a mean of 1.55 days and a standard deviation (SD) of 0.66 days (Lessler, et al. 2009). Only for influenza B, we assumed the shorter incubation period to yield a mean of 0.61 days and a standard deviation (SD) of 0.25 days.

medRxiv preprint doi: https://doi.org/10.1101/2024.08.17.24312064; this version posted August 19, 2024. The copyright holder for this preprint (which was not certified by peer review) is the author/funder, who has granted medRxiv a license to display the preprint in perpetuity.
This article is a US Government work. It is not subject to copyright under 17 USC 105 and is also made available for use under a CC0 license.

596 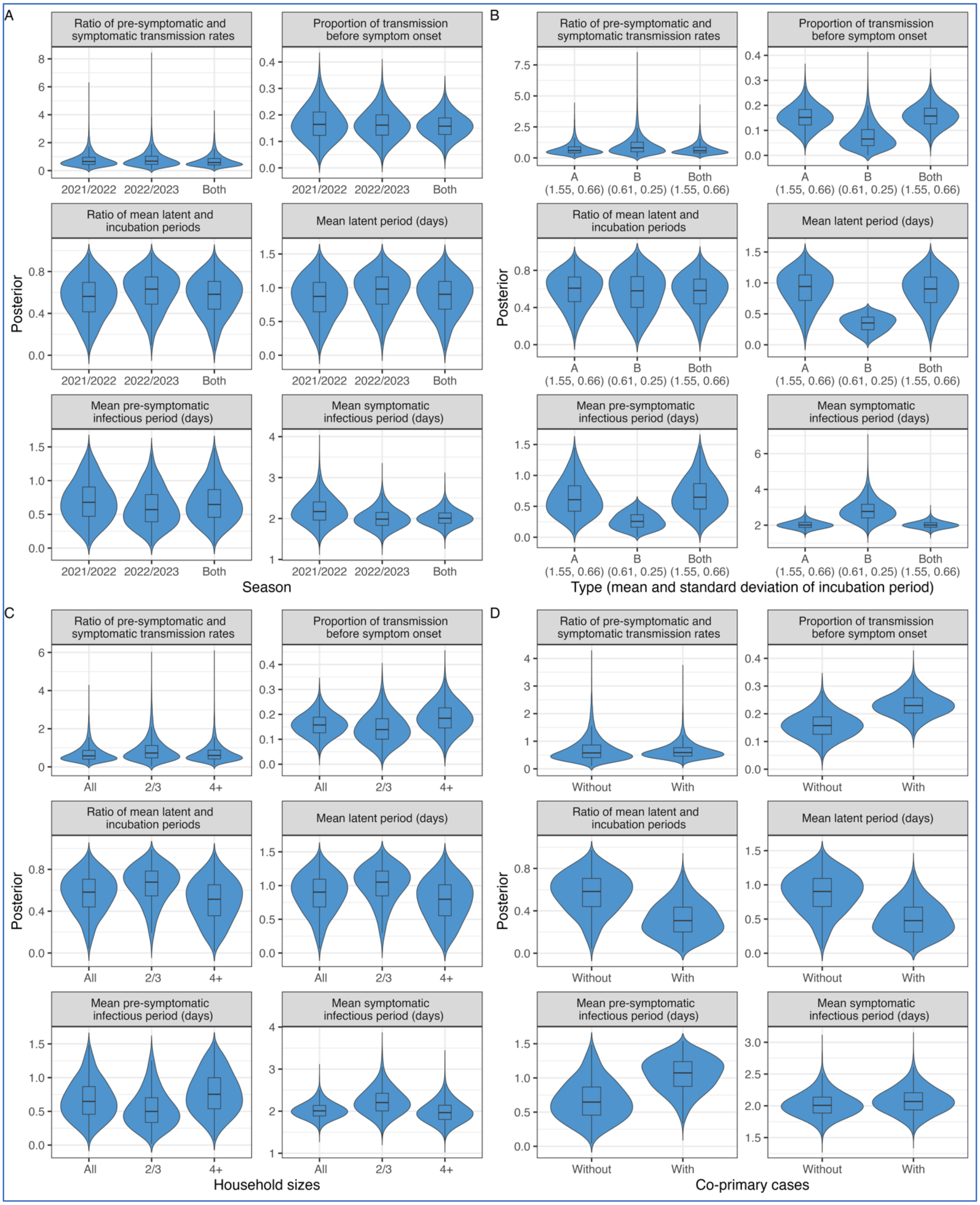

597 *Figure S3. Posterior distributions of parameters across data stratifications: (A) seasons, (B) virus types, (C) household sizes, and*
598 *(D) with multiple co-primary cases.*

medRxiv preprint doi: https://doi.org/10.1101/2024.08.17.24312064; this version posted August 19, 2024. The copyright holder for this preprint (which was not certified by peer review) is the author/funder, who has granted medRxiv a license to display the preprint in perpetuity.
This article is a US Government work. It is not subject to copyright under 17 USC 105 and is also made available for use under a CC0 license.

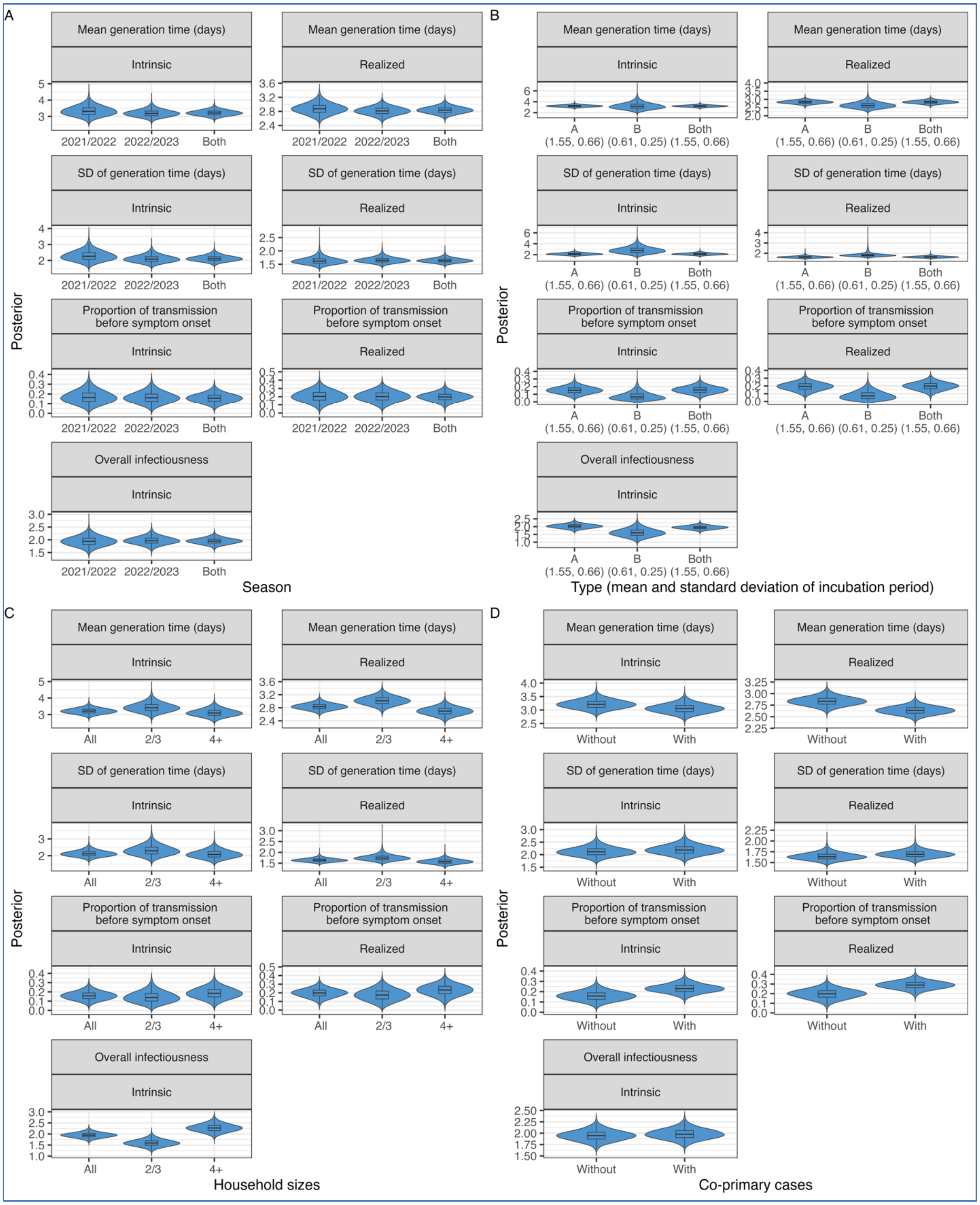

Figure S4. Posterior distributions of parameters across data stratifications: (A) seasons, (B) virus types, (C) household sizes, and (D) with multiple co-primary cases.

medRxiv preprint doi: https://doi.org/10.1101/2024.08.17.24312064; this version posted August 19, 2024. The copyright holder for this preprint (which was not certified by peer review) is the author/funder, who has granted medRxiv a license to display the preprint in perpetuity. This article is a US Government work. It is not subject to copyright under 17 USC 105 and is also made available for use under a CC0 license.

Sensitivity analyses

Similar to the sensitivity analyses using the full dataset, we found that the incubation period had a limited effect on the intrinsic generation time when exclusively using data from households circulating influenza A (Supplemental Figure S6, Panel B) or households circulating influenza B (Supplemental Figure S6, Panel C).

Consistent with the previous study (Hart, Abbott, et al. 2022), assuming a higher relative infectiousness of asymptomatic infected individuals resulted in slightly lower estimates of the overall infectiousness of infectors (Supplemental Figure S6, Panel D).

**Sensitivity analyses**	**Mean intrinsic generation time (95% CrIs)**	**Overlapping index (%, compared to the primary analysis)**
Primary analysis (in Table 1)	3.2 (2.9-3.6)	100
Longer incubation period	3.2 (2.8-3.6)	86
Shorter incubation period	3.4 (3.1-3.7)	56
Lower relative infectiousness	3.2 (2.9-3.6)	94
Higher relative infectiousness	3.2 (2.9-3.6)	97

Table S2. The posterior mean (95% CrIs) of mean intrinsic generation time given different incubation periods or relative infectiousness of asymptomatic infected individuals. The primary incubation period, derived from influenza A, had a mean of 1.55 days and a standard deviation (SD) of 0.66 days (Lessler, et al. 2009). For the shorter incubation period derived from influenza B, we assumed a mean of 0.61 days and a SD of 0.25 days (Lessler, et al. 2009). For the longer incubation period derived from influenza A(H1N1)pdm09, we assumed a mean of 0.61 days and a SD of 0.25 days (Tuite, et al. 2010).

medRxiv preprint doi: https://doi.org/10.1101/2024.08.17.24312064; this version posted August 19, 2024. The copyright holder for this preprint (which was not certified by peer review) is the author/funder, who has granted medRxiv a license to display the preprint in perpetuity.
This article is a US Government work. It is not subject to copyright under 17 USC 105 and is also made available for use under a CC0 license.

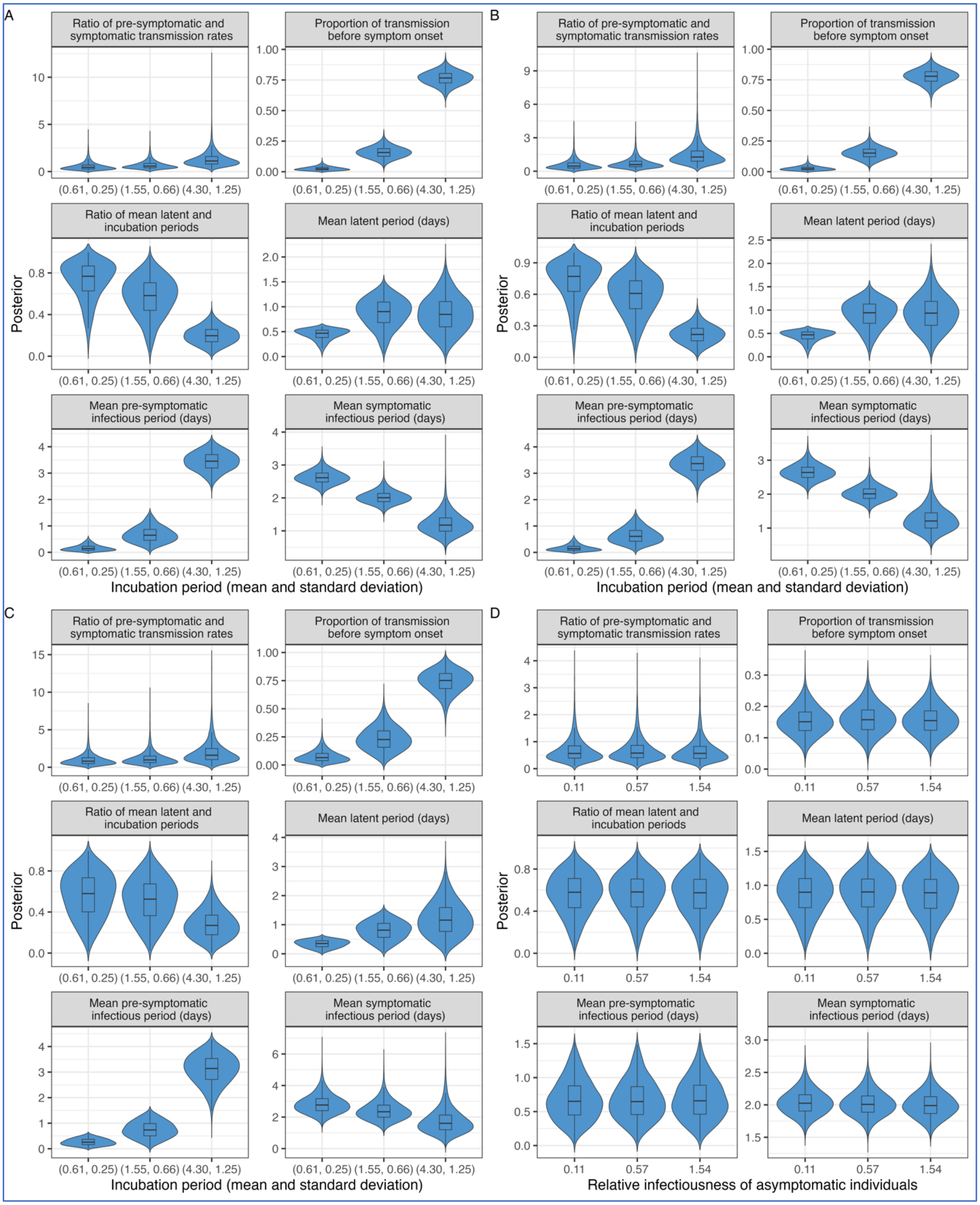

Figure S5. Posterior distributions of parameters given different assumptions: (A-C) incubation periods, and (D) relative infectiousness of asymptomatic infected individuals. Panel (A) presents results obtained using data from households with both influenza A and B, whereas Panels (B) and (C) present results obtained using data solely from households with influenza A and B, respectively.

medRxiv preprint doi: https://doi.org/10.1101/2024.08.17.24312064; this version posted August 19, 2024. The copyright holder for this preprint (which was not certified by peer review) is the author/funder, who has granted medRxiv a license to display the preprint in perpetuity.
This article is a US Government work. It is not subject to copyright under 17 USC 105 and is also made available for use under a CC0 license.

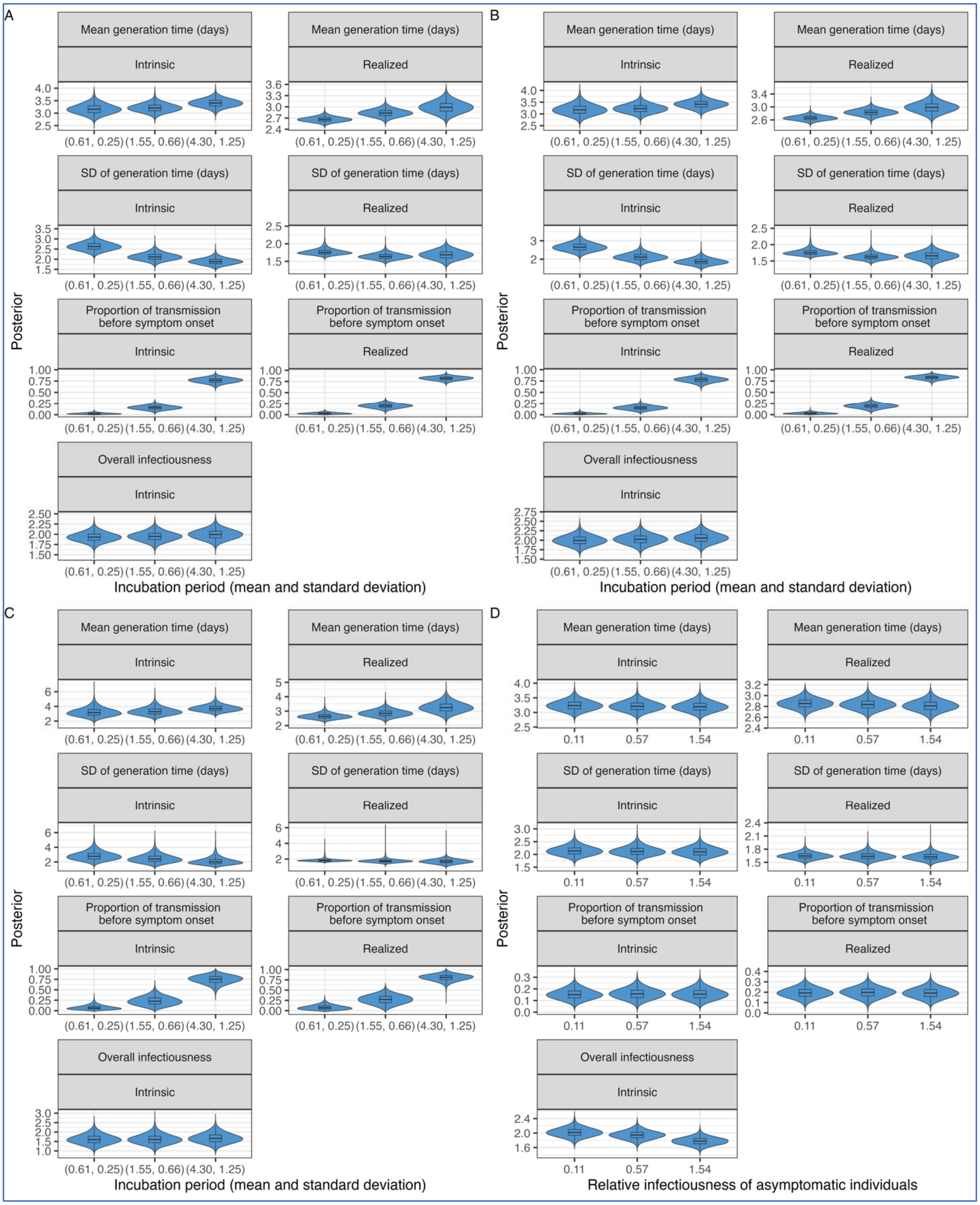

Figure S6. Posterior distributions of parameters given different assumptions: (A-C) incubation periods, and (D) relative infectiousness of asymptomatic infected individuals. Panel (A) presents results obtained using data from households with both influenza A and B, whereas Panels (B) and (C) present results obtained using data solely from households with influenza A and B, respectively.
